# Folded and Unfolded Conformations of Proteins Involved in Pancreatic Cancer: a Layman's Guide

**DOI:** 10.1100/tsw.2010.158

**Published:** 2010-08-17

**Authors:** Jerónimo Bravo, José L. Neira

**Affiliations:** ^1^ Instituto de Biomedicina de Valencia, CSIC, Valencia, Spain; ^2^ Instituto de Biología Molecular y Celular, Universidad Miguel Hernández, Elche (Alicante), Spain; ^3^ Instituto de Biocomputación y Física de Sistemas Complejos, Zaragoza, Spain

**Keywords:** pancreatic cancer, protein domain, structure, tyrosine kinase, disordered proteins

## Abstract

Pancreatic cancer (PC) is one of the most difficult illnesses to treat, since the 5-year survival rate is lower than 5% in patients and no substantial advances in its treatment have been achieved in the last 20 years. Since cancer deregulation and progression are associated with changes in at least one biochemical pathway, the knowledge of the structure of the proteins involved in such routes seems to be crucial in order to understand how this cancer progresses and, more importantly, to design more efficient and rationally designed drugs. In this review, we describe the fold and structures of proteins involved in different signaling pathways that intervene during PC development and progression. In particular, we will focus on the most frequently mutated, or alternatively differently expressed, proteins in PC. The current knowledge suggests that most of the proteins carry out their function by interacting with others via specific domains and through key residues at the recognition interfaces; these amino acids are mutated in patients developing PC. Furthermore, phosphorylation seems to be a crucial regulation step along several signaling pathways. Finally, we show not only that well-folded proteins in several signaling pathways are critical in the development of PC, but also that natively unfolded “hub” proteins, able to interact with DNA or proteins, are also important in such cancer progression.

## INTRODUCTION

Pancreatic cancer (PC) is the fifth most common cause of death in Western countries[1,2]. When it is detected, 90% of patients show metastatic infiltration in proximal lymph nodes, liver, or lungs. The current 5-year survival rate is roughly 2–3%[2,3]; in the best cases, when surgical removal is possible (roughly 10% of reported cases), the survival rate increases to 20%. Unfortunately, radiation and chemotherapy do not increase the rate of survival significantly. Mutations and deregulations of tumor suppressors (such as p53 and BRCA2), oncogenes, signaling molecules, and some cell surface receptors are known to be involved during the development of PC[4,5]. Although a great deal is known (see the accompanying papers in these series) about the specific events that take place during pancreatic carcinogenesis and the effect of several antibodies, limited structural information is available for most of the proteins involved in PC. The knowledge of the structure of such proteins is important in order to decide, in conjunction with their involvement in specific and key protein pathways, whether they can be considered as attractive therapeutic targets. The three-dimensional structure of target proteins is also essential to implement and develop a structural-based rational drug design strategy.

Signal transduction pathways intervene at different stages in the development of PC. Such pathways can be involved in tumor proliferation, resistance to apoptosis, invasion, metastasis, and angiogenesis. Reactivation of physiological embryonic signaling pathways is also frequent in PC. It has been proposed that signaling pathways altered in tumorogenesis or cancer progression represent promising potential targets for the development of future therapies. Therefore, in this review, we will focus on genes frequently mutated, or differentially expressed at any stage during the development of PC, that play an important role in the signaling pathways up-regulated in such illness. This approach can exclude some relevant proteins, such as p53[6], metalloproteases[7], or telomerases[8], which also intervene in PC[9,10], but they are not exclusive from PC and their structures have been extensively described elsewhere[6,7,11,12]. We will focus on the fold, domain architecture, and structure (when it is known) of individual proteins intervening in signaling pathways in PC, rather than on the signaling pathways themselves. We also describe the structure of the complexes formed with other interacting protein partners along the signaling pathway. The review is written for the layman, since we have attempted to convey all the terms clearly, trying not to introduce expert concepts on structural folds and protein domains, or when required for the description, the terms are explained first. The description of each protein has a brief introduction describing its importance in PC and the signaling pathway where it intervenes. Description of the structure of the isolated protein follows (when available) and, in some cases, the description of the complexes with other biomolecules along the corresponding signaling pathway, in order to show the importance of key residues at the recognition interfaces during PC development. We have divided the proteins described into two types: (1) those multidomain proteins whose structure, or that of their isolated domains, is known; and (2) those (a) proteins acting as natively unfolded "hubs" in protein networks and (b) whose three-dimensional structure has not been solved at the time of writing this review.

Our conclusions based on the gathered data suggest that (1) proteins involved or related with PC, and whose structures are known, are homo- or hetero-oligomeric species that are involved in complex signaling pathways; (2) when the structure of the complex between the protein and its target in a signaling pathway is known, mutations important in the development of PC appeared clustered around the recognition binding site, suggesting new clues to the development of more efficient, rationally designed drugs; (3) phosphorylation seems to be an important step for modulation of the activity of many of the proteins described and, interestingly enough, the tyrosine kinase domain is widely extended among the proteins involved in PC development, showing the importance of post-translational modifications and the use of kinases as possible therapeutic targets[13]; and (4) the proteins whose three-dimensional structures are solved are involved in several other malignant tumors, suggesting that the lack of PC-specific targets might be the reason for the limited effect of the current available therapies.

## PROTEINS WITH A KNOWN STRUCTURE

In the following, we shall describe the three-dimensional structure of proteins involved in PC development. In particular, we shall focus on some of the most frequently mutated or differentially expressed proteins in PC that are involved in up-regulation of signaling pathways.

Most of the proteins described below consist of several domains that fold independently. The most common domain found is tyrosine kinase. Due to its importance and since protein kinase pathways have been described as possible drug targets[13], we briefly describe this domain first (Fig. 1). It is structurally divided into two lobes. The amino-terminal lobe is formed almost entirely by β-strands that are covered on one side by an α-helix; the movement of this helical region is critical to acquire the active conformation

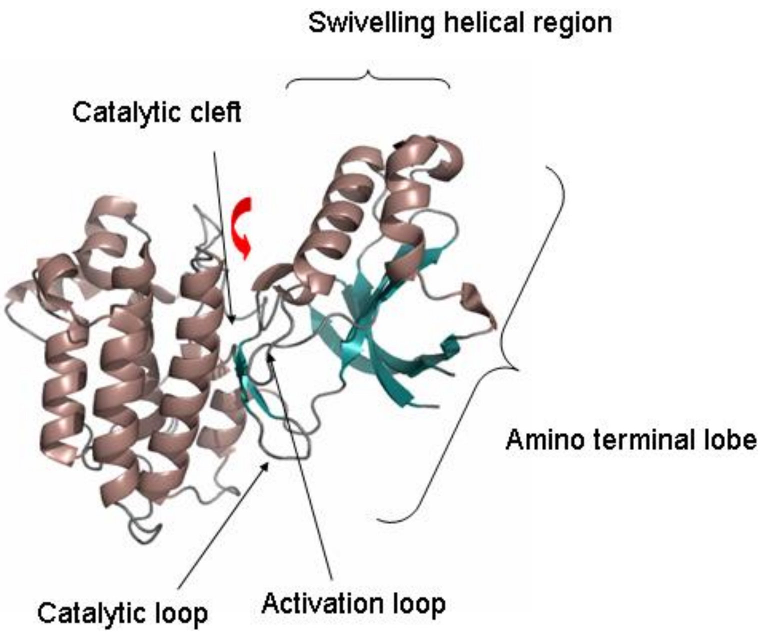

**FIGURE 1.** Structure of the protein tyrosine kinase domain. Tyrosine kinase domains are formed by two lobes with the catalytic cleft in between. The amino-terminal (right lobe) is mostly formed by β-strands. Helices are represented as ribbons and strands as arrows. Coordinates have been taken from PDB number 1R0P of an HGF receptor structure. The red arrow indicates the movement of the α-helix in the amino-terminal lobe, which occurs upon phosphorylation. This figure and those following have been created with PyMOL[126].

of the catalytic site. The C-terminal lobe is formed almost entirely by α-helices, with a short two-stranded β-sheet. Between the lobes, the catalytic cleft is located; this cleft is capped on one side by the catalytic loop and on the other by the activation loop; the latter is relocated by phosphorylation, and then it modulates the activity of the kinase.

## Hepatocyte Growth Factor Receptor (HGF Receptor Met Receptor)

The HGF receptor is an integral plasma membrane receptor tyrosine kinase overexpressed in 78% of PCs[14]. It is the receptor for HGF and the scatter factor; the HGF receptor is involved in cell proliferation, scattering, multicellular organism development, and survival. It is a proto-oncogene produced as a 1390-amino-acid single-chain precursor; the protein is cleaved by the cellular protease furin between residues 307 and 308 to yield a disulfide-linked two-chain heterodimer formed by two chains (of 50 and 145 kDa). The intact protein is formed by (1) a Sema domain (a variation of the β-propeller topology, see below, which is characterized by a set of conserved cysteines, forming four disulfide bridges); (2) a PSI domain (a cysteine-rich domain that is formed by a three-stranded antiparallel β-sheet and two α-helices); and (3) three, or four, IPT/TIG (immunoglobulin-like fold) domains and an intracellular domain that includes a protein tyrosine kinase domain. There are available structures of each isolated domain, which are described in the following.

The structure of the protein tyrosine kinase domain (residues 1049–1360) shows the well-known fold of protein kinases (see above)[15]. Kinase activation is achieved through autophosphorylation of tyrosines 1234 and 1235 in the activation loop (the so-called A loop). There is a conserved tandem-tyrosine polypeptide patch containing a multifunctional docking site at the C-terminus of the domain (the so-called supersite: $Y^{1349}$VHVNAT$Y^{1356}$VNV), which is absolutely required for signaling both *in vitro*

and *in vivo*. The first part of the motif (formed by Y[1349]VHV) has an extended conformation, whereas the second part (Y[1356]VNV), which is also a binding site for SH2 domains (see below the description of such domains), folds as a type-II β-turn. The ATY region of the supersite assumes a type-I β-turn conformation. The phosphorylated Tyr1349 and Tyr1356 residues serve as docking sites for a wide spectrum of transducers and adaptors, including PI3K, Src, Grb2, Shc, Gab1, and Stat3 proteins. The corresponding signaling complex triggers the intracellular downstream effects, including cell proliferation, scattering, and inhibition of apoptosis. There are also structures of the domain with organic compounds[15]; for instance, the furanosylated indolocarbazole K-252a, belonging to a family of microbial alkaloids that also includes staurosporine, inhibits autophosphorylation and is bound into the so-called adenosine pocket (formed, among others, by the following residues of the domain: Ile1084, Phe1089, Val1092, Ala1108, Lys1110, Leu1157, Met 1211, and the patch formed by Ala1226 to Tyr1230). The binding mode of alkaloid is analogous to that of staurosporine and, thus, this site can be considered a good target recognition site.

The three-dimensional structure of the c-Cbl tyrosine kinase binding (TKB) motif of the HGF receptor (residues 997–1009)[16] has also been solved. Cbl proteins appear to have two major physiological functions: acting as protein scaffolds or in targeting proteins for ubiquitination. c-Cbl interacts with a diverse array of proteins via (1) its phosphorylatable tyrosine residues located at its C-terminus, (2) its proline-rich region, and (3) its N-terminal phosphotyrosine-like binding domain, close to a RING domain. This phosphotyrosine binding domain structurally resembles an SH2 domain (see a structural description of the domain below) with an additional flanking four-helix bundle and a subdomain to accomplish binding. Together, these three subdomains make up the TKB domain, which is unique to Cbl proteins. Binding to the TKB domain of the HGF receptor is needed for the subsequent conjugation of ubiquitin; binding occurs between a phosphotyrosine and the conserved asparagine or the adjacent arginine of the TKB domain, directing the target polypeptide patch towards a positively charged pocket on c-Cbl.

The structure of the extracellular domain of HGF has also been solved (residues 25–740 of the intact HGF)[17]; this region includes the Sema domain, the PSI domain, and two IPT/TIG domains. The structure of the complex formed by this region and the surface protein of the human pathogen *Listeria monocytogenes* suggests that binding to the extracellular domain of HGF could occur through the first immunoglobulin-like and Sema domains; however, it is important to keep in mind that the cell surface protein of *Listeria* is not a structural mimic of HGF.

The structure of the isolated PSI domain (519–562) has also been solved by nuclear magnetic resonance (NMR)[18]. The structure represents a cysteine knot with short regions of secondary structure including a three-stranded antiparallel β-sheet and two α-helices. All eight cysteines are involved in disulfide bonds. It is suggested that the PSI domain could be a wedge between the propeller domain (Sema) and immunoglobulin (IPT/TIG) domains[18], and it seems to be responsible for the correct positioning of the receptor binding site in the Sema motif.

## Cholecystokinin B (CCK-B) and Gastrin Receptor

The CCK-B frequency of expression in PC is 95%. CCK-B is the receptor for gastrin (see below). In general, CCK-B receptors occur throughout the central nervous system, mediating its action by binding to G proteins[19,20]. There are three known isoforms. The largest, 516-residue-long isoform 2 is present in pancreatic and colorectal cancer cells, but not in normal pancreas or colonic mucosa[21].

The CCK-Bs are formed by several transmembrane (TM) domains, connected by cytoplasmic or extracellular loops, typically containing 50–80 residues. There are no structures of the whole protein, but there are three-dimensional structures in aqueous solution (NMR) in complex with other CCK regions and always within a lipid environment. For instance, the conformation of CCK(A)-R(1-47) (the fragment comprising residues 1–47 of the CCK-A) consists of a well-defined α-helix (residues 3–9) followed by a β-sheet stabilized by a disulfide bridge leading to the first TM α-helix[22]. The structure of the third extracellular loop (residues 352–379) of the human CCK-2 receptor, CCK2-R(352-379), consists of three helices, with the first and third helix corresponding to the extracellular ends of TM helices 6 and 7 of the whole protein, respectively[23]. The central helix (residues 363–368) is associated to the dodecylphosphocholine micelles used in the NMR studies. Upon titration of CCK-8 with the receptor domain, several NMR parameters show the formation of a stable complex and specific ligand/receptor interactions, suggesting the involvement of the TM helix 7. These results differ from other structural studies of CCK-8, where the association involved the TM helix 6. These differences might (1) play a role in the ligand specificity displayed by the different CCK receptor subtypes or, alternatively, (2) suggest the influence of the membrane mimetics used.

To conclude, the structure of the CCK/gastrin receptors, as inferred by the isolated polypeptide regions whose structures are available, appears to be formed mainly by α-helices.

## Sonic and Indian Hedgehog Proteins (Shh and Ihh)

The secreted morphogen Hedgehog (Hh) protein and its highly conserved homologs are involved in cellular differentiation during embryogenesis either in invertebrates or vertebrates. In mammals, there are three Hh genes that encode the Sonic hedgehog (Shh), the Indian hedgehog (Ihh), and the Desert hedgehog (Dhh) proteins. They are involved in processes such as neural tube, branching morphogenesis, bone formation, and spermatogenesis. Although Hh signaling is mostly inactive in adults, it has been implicated in many cancers. Shh is expressed in 70% of human pancreatic adenocarcinomas[24] and Ihh expression is increased 35-fold in PC cells compared to normal tissues[25].

In spite of having a metalloprotease-like protein fold, Shh acts as a ligand for membrane-bound receptors rather than as a protease. The TM protein Patched (Ptc1) is a negative regulator of the Hh pathway, which, in the absence of ligand, prevents Hh signaling by repressing the TM protein Smoothened (Smo). Binding of Shh to Ptc1 relieves the inhibition of Smo, allowing translocation to the primary cilium, where downstream signaling events lead to the activation of a family of zinc finger transcription factors. Smo is a G protein-coupled receptor protein that is conserved from flies to humans, and it can work as an oncogene. Hedgehog-interacting protein (Hhip, also known as Hip) is also a negative regulator of the pathway and it is up-regulated in response to Hh signaling, such as that occurring in the presence of Ptc1. The decreased expression of human Hhip has been noted in several tumor types, suggesting a potential role for Hhip in tumor suppression. The Shh protein contains two domains: the Hh amino-terminal signaling domain and the C-terminal Hint (Hedgehog/Intein) that has been split into N- and C-terminal regions to accommodate large insertions of endonucleases.

Structures of the Hh amino-terminal signaling domain isolated and forming complexes with several partners have been solved. The structures differ among the members of the family, but essentially contain a large antiparallel β-sheet[26], which can be covered on one side by two α-helices[27]. The structure of the Hhip in complex with Shh has also been solved. The Hhip is formed by two epidermal growth factor (EGF) domains (see the description of this domain below) and a six-bladed β-propeller domain (this domain is a type of all-β protein fold characterized by four to eight blade-shaped β-sheets arranged toroidally around a central axis). The three-dimensional structure shows that a loop of Hhip binds a groove of Shh where a $Zn^{2+}$ cation is present[28], coordinated by several histidine and aspartic residues. The comparison of Ptc1 sequences across several species reveals a sequence motif that is similar to this loop in Hhip, suggesting a similar way of inhibition of the Hh signaling and, thus, a function in tumor progression.

## Vascular Endothelial Growth Factor (VEGF)

Angiogenesis involves the growth of new blood vessels from pre-existing vessels, and tumors need a constant supply of nutrients through blood vessels. Angiogenesis is mediated in solid growth tumor by the VEGF family of proteins and receptors. The regulation of lymph vessel formation by VEGFs occurs through activation of three receptor tyrosine kinases (see above): VEGFR-1, -2, and -3. In addition, VEGF signaling is modulated through interactions with other coreceptors, such as heparan sulfate proteoglycans and neurophilins[29]. VEGFs have multiple isoforms that are generated by alternative splicing and post-translational processing, displaying distinct receptor specificities. Overexpression of VEGFs in PC is larger than 90%[30].

Six isoforms of VEGF are expressed in humans, having 121, 145, 165, 183, 189, or 206 residues in each monomer. The various isoforms have an N-terminal receptor binding domain in common, whereas the longer isoforms (having 165–206 residues) also include a 50-residue heparin binding domain at the C-terminal region. The structure of the heparin binding domain has been solved for $VEGF_{165}$ and it has two subdomains, each containing two disulfide bridges and a short two-stranded antiparallel β-sheet, where heparin is anchored[31,32]. VEGF binds to, and induces dimerization of, the tyrosine kinase receptors Flt-1 (fms-like tyrosine kinase-1) and KDR (kinase insert domain). Dimerization of KDR and Flt-1 occurs through symmetric binding to a pair of receptor binding sites, at the poles of the receptor binding domain of dimeric VEGF. Binding to KDR results in stimulation of vascular endothelial mitogenesis; on the other hand, binding to Flt-1 induces organizational effects on the vasculature. Structures have been published for several isolated VEGFs and/or their complexes with tyrosine kinase receptors, antibodies, or peptides[33,34,35,36]. Humanized neutralizing monoclonal antibodies against VEGF result in shrinkage of tumors; the binding sites of several such neutralizing antibodies have been shown to overlap with the KDR and Flt-1 binding sites on the VEGF receptor binding domain[37]. Antagonist peptides against VEGF also bind to the same regions as antibodies, KDR, or Flt-1[38]. The VEGFs whose structures have been reported to date are antiparallel, eight-residue-ring cysteine-knot polypeptide dimers that are covalently linked by at least two intermolecular disulfide bonds[39,40]; however, in mature VEGF-C, a mixture of covalently and noncovalently bound dimers has been described[41].

The common cysteine-knot domain of VEGF isoforms is formed by an antiparallel four-stranded β-sheet, three connecting loops (L1–L3), and an extended N-terminal α-helix (α1). Receptor specificity is determined by the N-terminal α-helix and the three peptide loops (Fig. 2), which form the receptor binding domain for dimeric VEGF. In some VEGFs, the cysteine-knot homology domain is flanked by C- and N-terminal propeptides, which are sequentially cleaved, giving rise to VEGF homologs with distinct functions.

## Src Proteins

Src is one of the members of the Src family kinases (SFKs) that are nonreceptor protein tyrosine kinases, reported to be critical for cancer progression; in fact, the Src family is one of the preferred targets for clinical development of new drugs (see [13] and references therein). Src is the product of the first proto-oncogene characterized[42] and it works as a protein switch. The output is the tyrosine phosphorylation and the input is the multiple protein-protein interactions occurring through several regions of its structure. Further, Src family members appear to respond to receptor-mediated signals, either by changes in kinase activity or by alterations in cellular location. Phosphorylated Src is inactive under healthy conditions, but 70% of PCs show the active form[43]. The main consequence of increased c-Src activity in tumor cells is to reduce cell adhesion, facilitate motility, and thereby promote an invasive phenotype. The c-Src kinase activity is an important component of the invasive phenotype in both early and advanced solid tumors. In early disease, c-Src kinase plays a key role in the epithelium-to-mesenchymal transition that marks the conversion of epithelial tumor cells to a more invasive phenotype. The increased c-Src kinase activity has been linked with the disruption of E-cadherin–mediated cell-cell adhesion, and affects the assembly and turnover of focal adhesions, which are critical for cell migration and cancer metastasis.

From a structural point of view, Src is a multidomain protein. It consists of an N-terminal SH3 domain followed by an SH2 domain. A tyrosine kinase catalytic domain is present at the C-terminus (see above). The SH2 and SH3 domains are involved in protein-protein interactions at several signaling cascades and they are also found in other families outside of the Src family (see above for other proteins

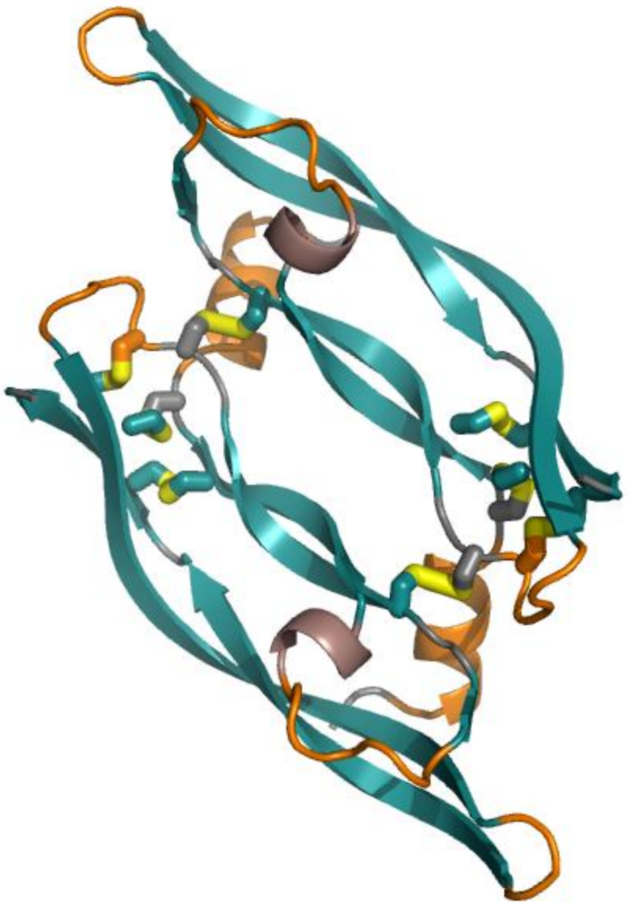

**FIGURE 2.** Structure of a cysteine knot. The VEGF structure (PDB number 1BJ1) shows the dimer of the antiparallel four-stranded β-sheet monomers covalently linked by intermolecular disulfide bonds depicted in yellow. Receptor specificity is determined by the N-terminal α-helix and the three loops shown in orange.

containing those domains). SH3 domains are involved either as isolated individual proteins or as portions of larger proteins, in signal transduction, and protein-protein recognition processes. The SH3 fold is composed of a compact β-barrel of five antiparallel β-strands. The five strands form two orthogonal β-sheets of three strands, with one strand shared by both sheets; at one of the ends of the strand, there is a small helical region (Fig. 3). Functional studies have shown that these β-barrel modules have a nonpolar groove complementary to peptides in a polyproline II conformation[44,45]. Furthermore, the proline-rich polypeptide patches in other proteins are also involved in interactions with SH3-containing modules. The SH2 domain consists of a β-sheet that is flanked on either side by two α-helices. SH2 domains bind polypeptide regions containing a phosphotyrosine, with a high specificity towards the residues following such a phosphotyrosine.

There is a structure available of a large fragment of a Src protein[46,47] in the phosphorylated and unphosphorylated forms, showing the switching mechanism of the protein. Briefly, phosphorylation of the tyrosine at the C-terminus creates an intermolecular binding site for the SH2 domain, locking the molecule in an inactive state; this movement concomitantly disrupts the kinase active site and sequesters the binding surfaces of the SH2 and SH3 domains. There are also several structures available for the individual SH3 domains[48].

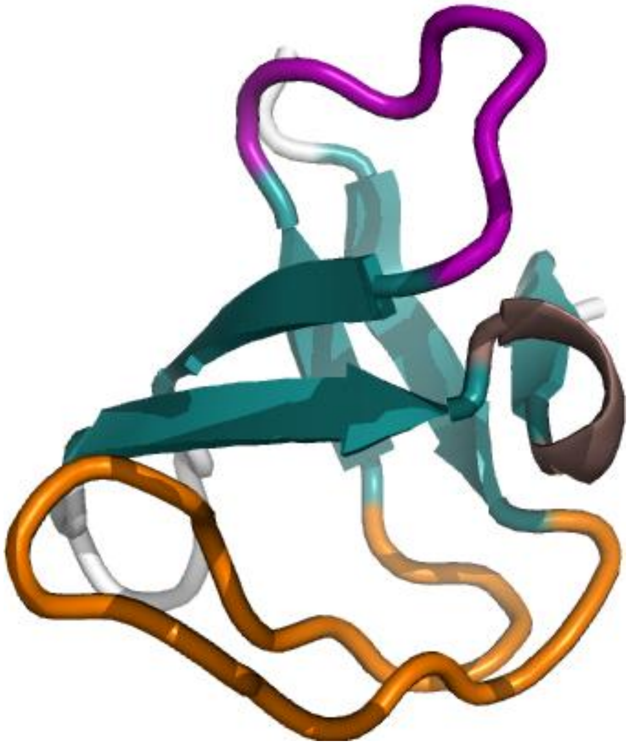

**FIGURE 3.** Structure of a SH3 domain. The structure of a SH3 domain from Src (PDB number 1FMK) shows the classical β-barrel of five antiparallel β-strands. Both RT loops connecting strands 1 and 2 (shown in orange), and the n-Src loops between strands 2 and 3 (shown in purple), are involved in recognition of proline-rich ligands.

## Epidermal Growth Factor Receptor (EGFR)

The human EGF receptor (EGFR) is a 1186-amino-acid-long TM glycoprotein. It is involved in the control of cell growth and differentiation. EGFR homo- or heterodimerizes with other members of the ErbB protein family and, thus, recruits intracellular proteins activating the signaling EGFR cascade[49,50]; the percentage of altered or overexpressed EGFRs in PC is between 20 and 50%[50]. It phosphorylates the protein Muc 1 in breast cancer cells and increases the interaction of Muc 1 with c-Src (see above) and β-catenin (see below)[51]. EGFs regulate cell differentiation and proliferation by binding to the EGFR extracellular region, with the resultant dimerization of the receptor tyrosine kinase domain. In fact, EGFR can be considered as a TM receptor tyrosine kinase with two domains. The extracellular portion is the 622-residue N-terminal region, which is divided into four articulated subdomains, I–IV, which are Furin-like repeats. Domains I and III share 37% amino acid identity, whereas domains II and IV are homologous Cys-rich domains. Domains I and III have the β-helix or solenoid topology expected from the sequence-related extracellular region of the insulin-like growth factor-1 (IGF-1) receptor[52] (see below for a structural description of this domain). The cysteine-rich domains II (amino acids 166–309) and IV (amino acids 482–618) contain a succession of small disulfide-bonded modules with an extended rod-like structure. Two types of disulfide-bonded module are seen in each domain. In one type (C1), a single disulfide bond constrains a bow-like loop. In the other (C2), two disulfide bonds link four successive cysteines to yield a knot-like structure. The domain II contains three consecutive C2 modules followed by five C1 modules. On the other hand, domain IV contains seven disulfide-bonded modules[53]. Finally, the intracellular domain of EGFR contains a protein tyrosine kinase domain.

Three-dimensional structures of EGFRs are available for the extracellular domain (residues 25–646) in complex with several ligands (see, e.g., [54]) and also for the isolated tyrosine kinase domain (residues 712–968)([55] and references therein). The structures of the monomeric or dimeric extracellular domain of EGFR[56] are not substantially altered upon binding to EGF; in fact, both ligand binding and dimerization occur through intramolecular domain arrangement of the Furin domains. For instance, in the monomeric form, the dimerization interface is occluded from the solvent by intramolecular interactions among domains II and IV[56], without changing the overall fold of each domain. Upon binding to EGF, the domains I–III of EGFR are arranged in a C shape, and EGF is docked between the Furin domains I and III. The 1:1 EGF-EGFR complex dimerizes through a direct swapped receptor-receptor interaction, in which a protruding β-hairpin arm of each EGFR domain II extends to hold the body of the other[57]. In that arrangement, the two cytoplasmic tyrosine kinase domains of the receptors are close enough for autophosphorylation and, thus, they activate the intrinsic tyrosine kinase activity. The EGFR tyrosine kinase domain is able to trigger numerous downstream signaling pathways, like other receptor tyrosine kinases and tyrosine kinase–linked cytokine receptors[58]. The extracellular region of EGFR is thought to modulate the spontaneous oligomerization observed in some cases when tyrosine phosphorylation occurs.

## β-Catenin Protein

The Wnt-β-catenin signaling pathway is involved in cell-cell adhesion, epithelial-to-mesenchymal transition, embryonic development, tumorigenesis, and regulation of angiogenesis. The pathway is down-regulated in adult organs, and alteration of the signaling cascade has a big impact in tumor progression and metastasis. Thus, accumulation of β-catenin is a hallmark of several cancers (and among them, PC, where accumulation of the protein occurs in 65% of the cases[59]). As β-catenin accumulates in the cell, it forms a complex with members of the Tcf family transcription factors and activates the transcription of several critical genes involved in cell proliferation. Then, disruption of such interactions should provide a way to treat several cancers.

β-Catenin is a 781-residue-long protein whose sequence contains several armadillo/β-catenin–like repeats. The three-dimensional fold of the protein shows that 12 repeats form a superhelix of α-helices with three helices per unit (Fig. 4) (where the 42-amino-acid sequence is the armadillo repeat unit). The cylindrical structure has a positively charged groove, at repetitions 5–9 of the armadillo motif, with two highly conserved lysines (the so-called charged “buttons”), where the Tcf transcription factors are anchored via two acidic residues. Mutation of either the lysines in the armadillo unit or the aspartic residues in the Tcf domain abolishes binding[60]. The crystal structure of β-catenin in complex with the N-terminal region of the Tcf3 domain from *Xenopus* showed that the complex has an elongated structure that extends along the positively charged superhelical groove formed by the β-catenin armadillo repeats[60] (Fig. 4). Binding in the Tcf3 transcription factor occurs through three binding modules: a β-hairpin module in the N-terminus, an extended central region that adopts a β-strand conformation, and a C-terminal α-helix. The structures of complexes of β-catenin with other Tcf transcriptional factors have also been solved and show a similar structural arrangement[61]. Interestingly enough, binding of the ICAT protein (a biomolecule that hampers the interaction between β-catenin and the Tcf family transcription factors) occurs through the groove formed by armadillo repeats 5–9 of β-catenin, but also via the 10–12 armadillo repeats[62], indicating that binding can take place at different positions of the armadillo structure.

## Focal Adhesion Kinase (FADK)

The FADK is a 1052-residue-long cytoplasmic multidomain protein involved in cell junction, cell motility, and survival; it is overexpressed in 48% of PCs[63]. Analysis of the primary structure of FADK shows that it contains an N-terminal ERM domain responsible for plasma membrane localization. The ERM

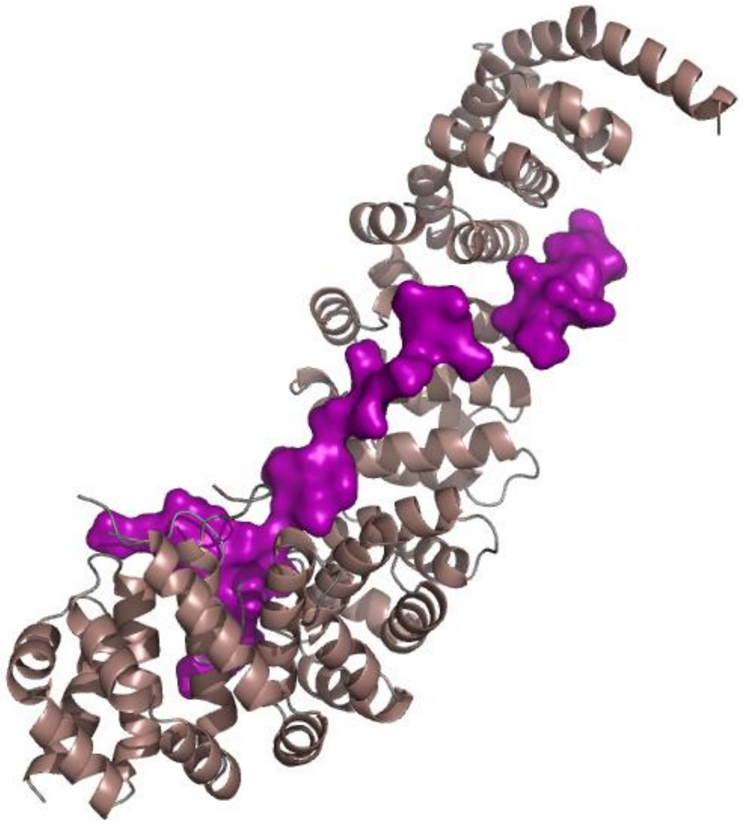

**FIGURE 4**. Structure of an armadillo repeat. The β-catenin structure (PDB number 1G3J) shows the superhelix of α-helices formed by 12 repeats of three α-helices. Binding of the Tcf3 catenin binding domain (shown in purple surface representation) takes place through a positively charged groove formed by the β-catenin armadillo repeats.

domain family is formed by ezrin, radixin, and moesin proteins (and so, the name of the family); each domain consists sequentially of (1) an N-terminal globular region, (2) an extended α-helical region, and (3) a highly charged C-terminal region, which in some proteins also encompasses a polyproline patch following the α-helical region. FADK also has a tyrosine kinase as catalytic domain and a focal adhesion targeting domain towards the C-terminus.

Structures are available for the focal adhesion targeting domain (residues 891–1052). Its overall structure is an antiparallel four-helix bundle with an up-down, up-down, right-handed topology[64,65]. There are short loop regions connecting this unusual parallel arrangement. The leucine-rich hydrophobic core of the domain also contains a methionine zipper from helices 1 and 4. These two helices are also tethered by two salt bridges (involving aspartic and arginine residues), but the helical bundle seems to be quite flexible since some crystal structures form swapped domains by intervening the N-terminal region and the first helix[65]. The Tyr925, which is phosphorylated by members of the Src kinase family, is located at the N-terminus of helix 1. The structure of the tyrosine kinase domain of FADK (residues 411–689) has also been solved and shows the described fold (see above)[66].

## AKT2 Protein

AKT2 or protein kinase B is a serine/threonine protein kinase in the PI3K-Akt pathway capable of phosphorylating several proteins and activating downstream targets such as mTOR or NF-κB. The AKT2

protein is activated by PI3K upon activation by Ras or, alternatively, by EGFR. It is overexpressed in 20% of PCs[67].

The analysis of the AKT2 primary structure shows an N-terminal Pleckstrin homology (PH) domain for binding to inositol phosphates and PI3K protein. It also contains a serine/threonine protein kinase catalytic domain; in fact, AKT2 phosphorylation at position Thr309 and Ser474 is required for full activity. An NMR structure for the PH domain and several X-ray structures for the serine/threonine kinase domain have been solved. The structure of the AKT2 kinase domain[68] shows a protein composed of two subdomains: one is a helical motif formed by eight to nine helical stretches, and the second is formed by a long antiparallel β-strand, on which three α-helices are packed. Both domains are connected by a flexible linker, where the catalytic cleft is located. The PH domain shows the same structure of other members of the PH family[69]. Its fold is formed by two perpendicular antiparallel β-sheets, followed by a C-terminal amphipathic α-helix. The lengths of the loops between the β-strands differ among the members of the family, providing the source of the domain specificity.

## The Cyclooxygenase (COX) Protein

Cyclooxygenases are bifunctional enzymes that catalyze the first step in the synthesis of prostaglandins, thromboxanes, and other eicosanoids. The initial COX reaction converts the achiral arachidonic acid to the chiral prostaglandin G2. There are two COX isoforms (COX-1 and COX-2), which share a high degree of amino acid sequence similarity, structural topology, and an identical catalytic mechanism. The expression of COX-2 is induced by tumor promoters, cytokines, and growth factors, and its expression is up-regulated in 90% of PCs[70]; conversely, COX-1 is constitutively expressed and has a homeostatic role. The mechanisms modulating cancer development through COX involve (1) several mitogenic signaling pathways and (2) interaction with other molecules mediating resistance to apoptosis, angiogenesis, and immunosuppression[71].

Both isoforms are 600-residue-long proteins that form homodimers, where only one subunit of the homodimer is active at a time during the enzymatic reaction[72]. Both proteins are α-helical (formed by up to 69 helical regions, Fig. 5) connected by short strands of a twisted β-sheet[73,74]. The site for the fatty acid in all the structures of complexes with either isoform is located in the so-called COX channel (Fig. 5). In such a channel, the carboxylate of the substrate interacts with the guanidinium group of an arginine residue (this residue is a key determinant for binding of the fatty acid to COX-1, but not for COX-2[75]), and its end region binds to a hydrophobic groove that is capped by a tyrosine residue.

## Insulin-Like Growth Factor I (IGF I) Receptor

The IGF I receptor is a TM protein with tyrosine kinase activity that plays an important role in cell growth control. The IGF I is overexpressed in 64% of PCs[43] and its subunit structure is formed by a tetramer of two α- and two β-chains linked by disulfide bonds. The α-chains contribute to the formation of the ligand binding domain, whereas the β-chains contain the tyrosine kinase domain.

The extracellular region of IGF I is formed by two leucine-rich repeats (forming a right-handed β-α superhelix) with a cysteine-rich domain in between (L1-Cys-rich-L2); each of the L domains consists of a single-stranded right-handed β-helix, similar to the fold described for the extracellular domain of EGFR (see above). The three domains surround a central space of enough size to accommodate a ligand molecule. The extracellular region is completed with three fibronectin type-III domains (which have a β-sheet sandwich motif). The intracellular portion holds the catalytic tyrosine kinase domain. Thus, it can be concluded that the overall disposition of the domains in the extracellular region of IGF I is similar to that observed in the EGFR.

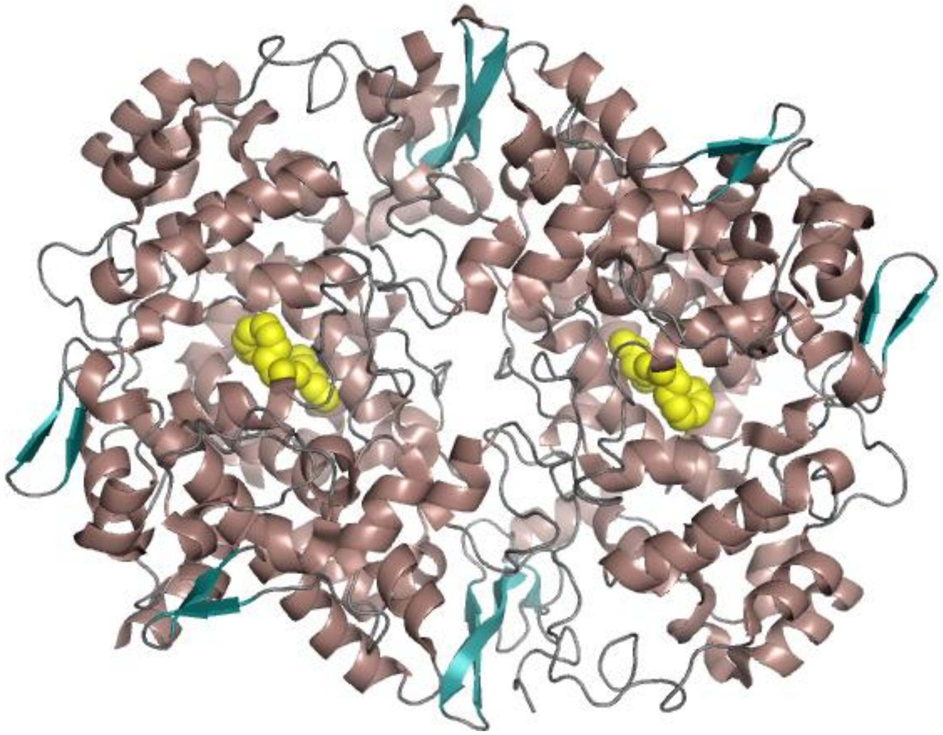

**FIGURE 5.** Structure of a COX-2 protein. The COX-2 protein is mainly an α-helical homodimer (PDB number 1CVU). The helical regions are connected by short strands of a twisted β-sheet. The arachidonic acid, in the active site, is shown as yellow spheres.

Most of the available structures of IGF I describe the tyrosine kinase domain (see above). There is also a structure of the three N-terminal domains (L1-Cys-rich-L2)[76], having the cysteine-knot fold described above for the C2 Cys-rich domains of the extracellular domain of EGFR.

## SMAD Family Member 4 (Smad4)

Smad4 is a 550-residue-long protein that acts as a common mediator of signal transduction by the TGF-β (transforming growth factor-β) superfamily of cytokines. After receptor kinase activation, the signals of the TGF-β family are regulated by several evolutionarily conserved proteins known as SMAD proteins; several SMAD proteins have been characterized in vertebrates. The Smad4 member, also known as DPC4 (deleted in pancreatic carcinoma locus 4), has a central role in signal distribution by forming hetero-oligomers with other pathway-restricted SMAD proteins: Smad1, 2, 3, 5, and 8, which function in specific signaling pathways. These pathway-restricted SMAD proteins are phosphorylated at the conserved C-terminal tail sequence, $SS^{p}XS^{p}$ (where the $^{\text{“p”}}$ denotes a site of phosphorylation), by a receptor kinase, in response to TGF-β activation. The heteromeric complexes of Smad4 with the phosphorylated SMAD proteins are translocated into the nucleus. In the nucleus, the heteromeric complexes function as gene-specific transcription activators by binding to promoters and interacting with transcriptional coactivators. Defects in Smad4 are a cause of PC[77] and mutations of the protein cause a gastrointestinal polyposis, which can develop gastrointestinal cancers.

Smad4 is a multidomain protein. It contains an N-terminal DWA (domain A in the dwarfin protein family) and a C-terminal DWB (domain B in the dwarfin protein family) domain (also called MH1 and MH2 domains) separated by a variable proline-rich linker region (the so-called L domain); in general, all SMAD proteins share that common domain configuration.

The crystal structure of the MH1 domain for several pathway-restricted SMAD proteins shows a globular fold consisting of four α-helices (α1–α4) and short β-strands (β1–β6) connected by loops. The 11-residue-long β-hairpin formed by strands β2 and β3 protrudes from the core of the otherwise globular MH1 domain, and it is inserted into the major groove of the bound DNA. Recent reports of several pathway-restricted SMAD proteins suggest that there is not a generally recognized DNA consensus sequence[78,79,80].

The crystal structure of the Smad4 MH2 domain (residues 319–552) suggests that the functional unit of SMAD proteins is a trimer species. The MH2 domain forms a crystallographic trimer, through a conserved protein-protein interface, to which the majority of the tumor-derived missense mutations map[81]. Each monomer is formed by five α-helices and three loops enclosing a β-sandwich. Mutations of the residues comprising residues 506–522 (one of the loops) disrupt the heteromeric interaction between Smad4 and the other pathway-restricted SMAD proteins, suggesting that protein-protein interactions occur via such a highly conserved loop; interestingly enough, several tumor-derived mutations are also located in that region[81]. Moreover, this loop is also involved in the interactions of Smad4 with the members of the Ski family of proto-oncoproteins[82]. An additional SMAD-activation domain (SAD) is located at the N-terminal of the MH2 domain[83,84]. In the X-ray structure of the fragment comprising the SAD and the MH2 region of Smad4, the SAD region appears to form the core of the protein[85], with a large solvent-exposed proline-rich region. This region, together with a nearby glutamine-rich helix, is proposed to be a potential transcription activation surface[85].

## p16 or INK4 Protein p16 is a 150-residue-long protein whose overexpression causes cell cycle arrest and inhibition of tumor cell proliferation in cell cultures[86]. The p16 protein functions by inhibiting the activity of cyclin-dependent kinase 4 (cdk4) or cdk6. When inhibited, cdk4 and cdk6 cannot phosphorylate several regulatory proteins involved in the cell cycle; thus, inactivation occurs by blocking the association of cdk4/6 with cyclin D, preventing kinase activation and resulting in cell cycle arrest by p16[87]. This inactivation contributes to a variety of neoplasias and, interestingly enough, p16 mutants (which lack the ability to arrest cell growth) have been found in more than 70 different types of tumor cells[88,89] and they are frequently observed in PC[90].

The structure of p16 is characterized by a four-linear (I–IV) array of a repeating structure: the so-called ankyrin motif[91]. Each 33-residue-long ankyrin repeat exhibits a helix-turn-helix (H-T-H) fold (with an overall shape resembling an "L"), although the first half of the second ankyrin repeat of p16 consists of only one helical turn; all the helices of the motif are packed into helical bundles. The four H-T-H motifs are connected by three loops whose orientations are such that they are perpendicular to the helical axes; in that arrangement, the ankyrin repeats stack to yield a concave surface on one side (in fact, this surface is formed by the entire ankyrin repeat III, the third loop and the N-terminal region of the fourth helix in the second repetition). Mutations involved in several PCs appear more frequently in the second loop. This region is also the one involved in binding to cdk6[92], where the recognition between the two proteins is mediated primarily by hydrogen-bond networks and, thus, the overall structure of p16 is not altered upon binding (as happens in EGFR upon recognition to their corresponding partners, see above).

## K-Ras Protein

The K-Ras is a 189-residue-long protein that binds GDP/GTP and possesses intrinsic GTPase activity. In general, Ras proteins transmit extracellular signaling from receptor tyrosine kinases to two serine/threonine kinases (Raf and MEK) and, finally, lead to the activation of mitogen-activated protein kinases (MAPKs). Upon nuclear import, MAPKs phosphorylate many different transcription factors, modulating DNA binding affinity, nuclear localization, and thereby regulating gene expression. Several studies demonstrate that Ras/MAPK signaling plays a role in normal development[93,94,95]; moreover, between 15 and 20% of all human tumors have an activating mutation in one of the three *ras* genes (N-, K-, or H-*ras*), which code for very similar proteins of around 21-kDa molecular mass. These proteins are post-translationally modified by the covalent attachment of lipophilic groups to the C-terminus; this modification targets Ras proteins towards the plasma membrane, where their interacting proteins are found[96]. Although many of the components of Ras/MAPK signaling have been characterized, the full array of transcription factors affected is not known; moreover, the detailed mechanisms by which phosphorylation modulates transcription factors is still unclear in many cases. The lifetime of the signal transduced by Ras proteins is determined by the lifetime of the GTP-bound state. These GTP binding proteins work as molecular switches by cycling between GDP- and GTP-bound states; being the exchange of the bound GDP for GTP facilitated by guanine-nucleotide exchange factors. Mutant forms of the human Ras genes (at positions 12, 13, and 61) that yield a protein with a prolonged GTP-bound state (due to a smaller rate of GTP-hydrolysis) are found in human cancers[97,98].

The K-Ras protein appears as two isoforms that differ at the C-terminal region. Mutations in K-Ras are behind the onset of different leukemias, and those mutations are also involved in the development of bladder, stomach, colon, cervix, brain, pancreatic, and lung cancers[93,94,95,99,100,101]. The X-ray structures of several Ras proteins have been solved (see, for instance, [97,100] and references therein). The structure is composed of a six-stranded β-sheet, which is packaged on one side by a five α-helical bundle. The GTP binding site involves one of the helices and a long loop connecting with a β-strand; the structural difference between the GDP- and GTP-bound states involves an increase of the length of an α-helix which is set perpendicularly to the binding site in the GTP-bound state[100,102,103,104,105,106].

### Notch 3 Protein

Notch 3 functions as a receptor for membrane-bound ligands Jagged 1, Jagged 2, and Delta 1 to regulate cell-fate determination. It is expressed in around 70% of PCs[107]. Notch 3 is involved in the Notch signaling pathway; it forms a transcriptional activator complex and activates several genes upon ligand activation through the released notch intracellular domain. In general, Notch 3 affects the implementation of differentiation, proliferation, and apoptotic programs.

Structurally, Notch 3 is a heterodimeric complex of C- and N-terminal fragments, which are probably linked by disulfide bonds. There are no three-dimensional structures available of Notch 3, although the structure of Notch-1-ankyrin domains and EGF domains have been solved ([108] and references therein), and it will be described in the following. At the N-terminal extracellular portion of Notch 1, there are several repetitive EGF domains and three NL domains (a domain found in Notch and Lin-12 proteins with three disulfide bridges and three conserved aspartate and asparagine residues). The EGF domain has three disulfide bridges and is formed by a two-stranded β-sheet followed by a loop that joins to a short two-stranded β-sheet at the C-terminus. Notch 3 also contains six ankyrin repeats in the C-terminal fragment (see above for a description of ankyrin motifs).

## PROTEINS WITH AN UNKNOWN STRUCTURE OR NATIVELY UNFOLDED

In the following, we shall describe the few structural data that are known on the structure or fold of other proteins involved in signaling pathways during the development of PC. The three-dimensional structures of those proteins are not known because the proteins involved are natively unfolded or, alternatively, because at the day of writing this review, and although they can show a sequence similarity to proteins whose structure is known, there are no detailed structural reports on the particular protein described.

## Pancreatitis-Associated Protein

Induction of acute pancreatitis in rats showed the presence of a new protein in the pancreatic juice during the early stages of the illness; the protein was overexpressed in the following 3–4 days[109] and then it was called pancreatitis-associated protein (PAP). PAP is constitutively expressed in the healthy pancreas by the α-cells of Langerhans islets[110] and it has been involved in the response of pancreatic cells to cell injury (see [111] for a detailed overview of the different functions of PAP). Interestingly enough, PAP mRNA expression was observed in pancreatic and hepatocellular adenocarcinomas and some of the mucinuous cystadenomas[112,113].

PAP is a 175-residue-long polypeptide containing a carbohydrate binding domain (CBD)[112,113], which is the binding motif of C-type $Ca^{2+}$-dependent lectins. At the N-terminus of PAP, there is a short peptide that is cleaved during maturation[114]. Sequence similarities between PAP and other lectin domains range from 16 to 26%, and even the gene organization of PAP suggests that the protein belongs to a new type of lectin that has evolved from the same carbohydrate-recognition domain[115]; furthermore, PAP only contains the CBD, conversely to what happens to other lectins where additional motifs are present and provide the specific functions of lectins. Attempts to characterize a carbohydrate-binding affinity in PAP have failed[116] and no structural approach has been tackled. Thus, due to the high structural diversity among the C-type lectins (from immunoglobulin-like folds to coiled coils), no clues about PAP structure are known.

## The Stress-Inducible p8 Protein

The p8 (also called NUPR1 [nuclear protein-1] or Com1 [candidate of metastasis-1]) was first described as a gene induced during pancreatitis in pancreatic acinar cells[117]. The protein is overexpressed in several cancers, acting as a cell-stress response (see [118] for a recent review on p8 functions).

High-resolution structural studies by NMR, fluorescence, and CD spectroscopies have shown that p8 is disordered[119]. Moreover, analysis of the sequence with several recent disorder predictors indicates that p8 belongs to the class of "natively unfolded proteins" (Fig. 6). The primary structure of p8 shows a large abundance of arginine, proline, serine, and glycine residues, and an almost complete absence of disorder-promoting residues, such as leucine or valine, when compared to the average propensity of these residues in the PDB structures (Fig. 6A). The use of Foldindex[120] indicates that regions comprising residues Ala3 to Thr9, and Ser32 to Tyr37, are the only ordered polypeptide patches. On the other hand, the RONN predictor[121] suggests that p8 is completely disordered, although the region from Asp29 to Arg43 appears less disordered than the rest of the polypeptide patches (Fig. 6B). Thus, p8 is the first protein involved in PC that has been identified as a "natively unfolded protein".

The protein is able to interact with other proteins or DNA. The phosphorylated p8 interact with chromosomic DNA[119]. On the other hand, p8 binds to the hystone acetyltransferase-associated protein MSL1, contributing to chromatin rearrangement by facilitating the access to DNA of the transcription machinery[122]. The p8 protein also interacts with the antiapoptotic protein prothymosin-α, suggesting that p8 has nontranscriptional roles[123]. The p8-prothymosin-α complex is able to inhibit the staurosporine-induced apoptosis. Structurally, binding occurs in regions close to the two tyrosine residues of p8 (Tyr31 and Tyr37), and at least one of the two proteins seems to acquire a folded structure. However, it is not known whether the folded-upon binding regions involve the whole sequence or, alternatively, the acquired structure is highly localized, leading to a rather disordered, "fuzzy" complex[124].

## Gastrin Precursors and Gastrin Proteins

Gastrin precursors and fully amidated gastrin are expressed in 80 and 25% of PCs, respectively[125]. Gastrin is a peptide hormone that is secreted by the G cells in the gastric antrum and duodenum. Gastrin

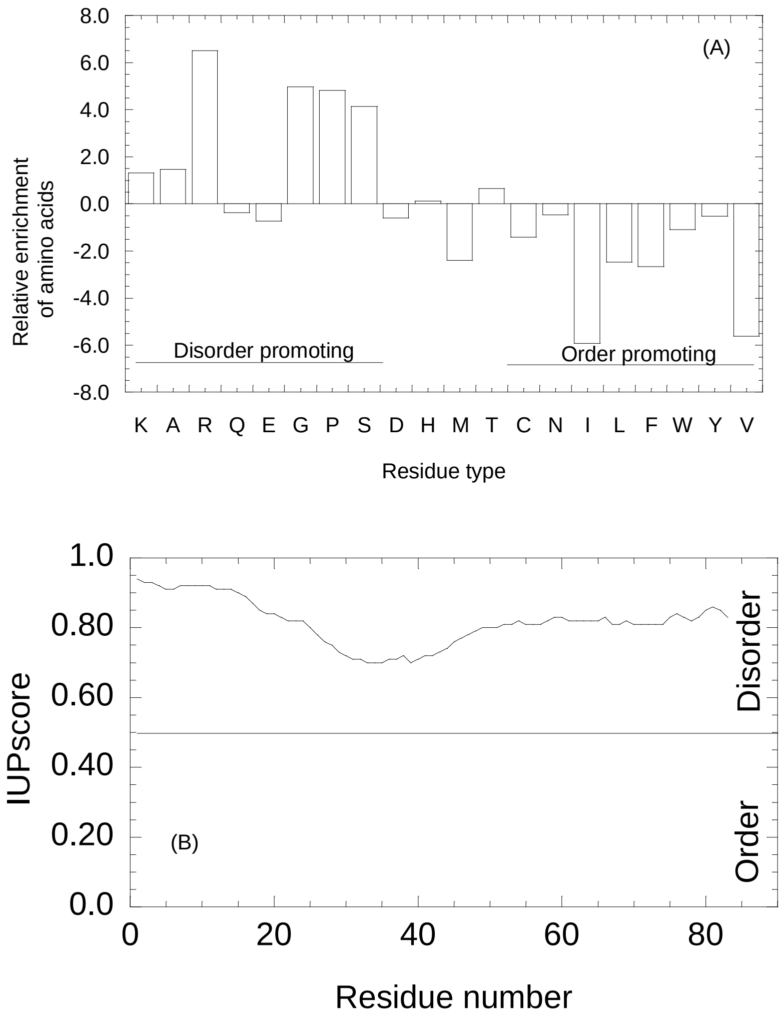

**FIGURE 6.** In *silico* analysis of p8 sequence. (A) Distribution of order- and disorder-promoting amino acids in the p8 sequence. The distribution shows the deviation in amino acid composition of the domain from the average values in the Swiss-Prot database (as obtained from http://www.expasy.org/sprot/relnotes/relstat.html ). (B) RONN prediction of unstructured regions of p8. The threshold is also 0.5. The p8 sequence is: GSATFPPATSAPQQPPGPEDEDSSLDESDLYSLAHSYLGGGGRKGRTKRRAAANT NRPSPGGHERKLVTKLQNSERKKRGARR.

acts on the stomach mucosa and on the pancreas to activate secretion of digestive enzymes. Due to its small size (polypeptide lengths range from 17 to 34 amino acids out of the 108 residues of the precursor), there are no reported structural data.

## CONCLUSIONS

We have reviewed the conformational propensity of unfolded proteins and the fold, domain scaffolding, binding partners, and the structure, when available, of those proteins known to intervene in PC and involved in signaling pathways. Our description shows that the majority of the proteins are multimeric. The domains serve as (1) anchor points to other biomolecules or (2) phosphorylation sites for protein networks. These results suggest that protein networks are important to fully understand PC and that phosphorylation seems to play key roles in PC development.

Furthermore, the binding to the corresponding protein or biomolecule by the described protein does not alter substantially the overall topology of the isolated proteins, involving only small rearrangements around particular side chains of residues. These results pinpoint the (1) importance of key residues in PC by altering a signal pathway, since the resulting mutants, due to the different side chain of the mutated amino acid, can hamper proper docking of the target; and (2) use of structural models to design rationally more effective drugs. The amount of data gathered also reinforces the importance of protein networks, not only by the existence of key residues at selected proteins in the signaling pathway, which may govern protein-biomolecule interactions, but also because of the detection of “hub” proteins that are natively unfolded proteins at crucial points of such networks. Finally, the findings reviewed here indicate that the majority of the proteins involved in PC intervene in other types of cancers (since they are involved in general signaling pathways), suggesting the absence of specific protein targets for this cancer. This observation might be related with the absence of a specific treatment for PC. However, most of the signaling pathways up-regulated in PC contain proteins with tyrosine kinase domains that might be of therapeutic interest either isolated or by a combined use of future inhibitors that simultaneously target several pathways.

## ACKNOWLEDGMENTS

We deeply thank both reviewers for their comments and discussions, and Prof. Juan Iovanna for suggestions of the addition of some proteins to widen the scope of this review. The work of JB in the laboratory was supported by the Ministerio de Ciencia e Innovación (SAF2008-04048-E, SAF2009-10667), EVES AP001/10, and CSIC (200820I020). The work of JLN in the laboratory was supported by the Ministerio de Ciencia e Innovacion (SAF2008-05742-C02-01, CSD2008-00005), Generalitat Valenciana (ACOMP2010/114), and FIPSE Foundation (Exp: 36557/06). We deeply thank May García, María del Carmen Fuster, Javier Casanova, and Raquel Jorquera for assistance.
